# The Expression of Emotions in 20th Century Books

**DOI:** 10.1371/journal.pone.0059030

**Published:** 2013-03-20

**Authors:** Alberto Acerbi, Vasileios Lampos, Philip Garnett, R. Alexander Bentley

**Affiliations:** 1 Department of Archaeology and Anthropology, University of Bristol, Bristol, United Kingdom; 2 Centre for the Study of Cultural Evolution, Stockholm University, Stockholm, Sweden; 3 Department of Computer Science, University of Sheffield, Sheffield, United Kingdom; 4 Department of Anthropology, Durham University, Durham, United Kingdom; Technical University of Denmark, Denmark

## Abstract

We report here trends in the usage of “mood” words, that is, words carrying emotional content, in 20th century English language books, using the data set provided by Google that includes word frequencies in roughly 4% of all books published up to the year 2008. We find evidence for distinct historical periods of positive and negative moods, underlain by a general decrease in the use of emotion-related words through time. Finally, we show that, in books, American English has become decidedly more “emotional” than British English in the last half-century, as a part of a more general increase of the stylistic divergence between the two variants of English language.

## Introduction

Studies of cultural change and evolution have been transformed recently by a new level of accessibility and volume of electronic data concerning human behavior [1]. Among these, new studies of word usage can access mass everyday public preferences that may be missed in mainstream history and politics [2]–[8]. For example, interest in mass public ‘mood’ was sparked by claims that Twitter mood words can predict stock market trends [9], and, in general, sentiment analysis of Twitter stream is a growing field of research [10]–[12]. This has been paired by findings of surprisingly straightforward patterns about word usage online, such as S-curves of adoption [13]–[15], or traders whose instant messaging patterns correlate with their financial success [16]–[18].

While these studies focus on the recent, short-time scale of online media, one of the exciting challenges of the ‘Big Data’ agenda is to address cultural dynamics at longer time scales. Language itself is a remarkably long-lived phenomenon, with most of the common words in a language having been passed down through multiple generations for centuries or millennia [3], [4], [19]. While studies of word usage in different online communities [13] can achieve unprecedented sample sizes, long-term temporal dynamics and diversity are exhibited by the history and inferred prehistory of human languages [20]–[23].

In a novel approach, Hughes et al. [7] investigated past centuries of books (using the Project Gutenberg Digital Library) through the frequencies of ‘content-free’ words, that is words that carry little or not meaning on their own, but acquire it according to the context in which they are used, such as “to be” verbs, conjunctions (e.g. *and*, *but*), articles (e.g. *the*), pronouns (e.g. *you*, *us*) and prepositions (e.g. *about*, *within*). Representing authors by a vector of normalised content-free word frequencies used in their works, Hughes et al. [7] found temporal regimes of similarity among authors (1784–1829, 1825–1870, 1866–1911, and 1907–1952) that provided a fascinating comparison with well-known historical genres of literature. In evolutionary terms, words with specific content are selected to serve a more direct purpose, but the content-free words subject to random drift mark differences in stylistic genre.

If content-free words are a good proxy for stylistic change, we may add in increments of ‘content’ and see how patterns change. For example, using Google's Ngram database [2], Twenge et al. [8] found that the summed frequencies of ‘individualistic’ words (e.g., *independent*, *individual*, *unique*, *self*, *solitary*, *personal*) significantly increased in American books between 1960 and 2008, while ‘communal’ words (e.g., *communal*, *team*, *collective*, *village*, *group*, *union*) did not. During those same 48 years, individualistic phrases (e.g., *all about me*, *I get what I want*) also increased in frequency compared to communal phrases (e.g., *band together*, *unitedi we stand*) [8].

Here we analyze trends in the past century of mood words in books, using Google's Ngram database. Google's Ngram database represents a 4% digitally–scanned sample of several centuries of books, for a total of 5,195,769 volumes [2]. The corpus contains texts in different languages, and, for English, a further distinctions is made between American English and British English (according to the country of publication, i.e. United States versus Great Britain). Additionally, a subset of English texts collects only fiction books. Titles of books present in the corpus are not available because of copyright reasons [2]. The corpus gives information on how many times, in a given year, an 1-gram or an n-gram is used, where an 1-gram is a string of characters uninterrupted by space (i.e. a word, but also numbers, typos, etc.) and an n-gram is a sequence of *n* 1-grams.

We make use of six unique lists of terms (see Methods) to characterize mood categories labeled as Anger, Disgust, Fear, Joy, Sadness, and Surprise. These mood word lists have previously been applied on a study of U.K. Twitter content, which showed that changes in these mood word frequencies identified real-world events such as the unexpected deaths of popular personas, public unrest, or natural disasters [24]. We extend the time scale of this analysis by tracking mood word frequencies through the past century of Google book data. We find a general decrease in the use of mood terms through time, which underlies a distinct increase in emotional word usage in American books versus British books in the last half century.

## Results

**Figure 1:**
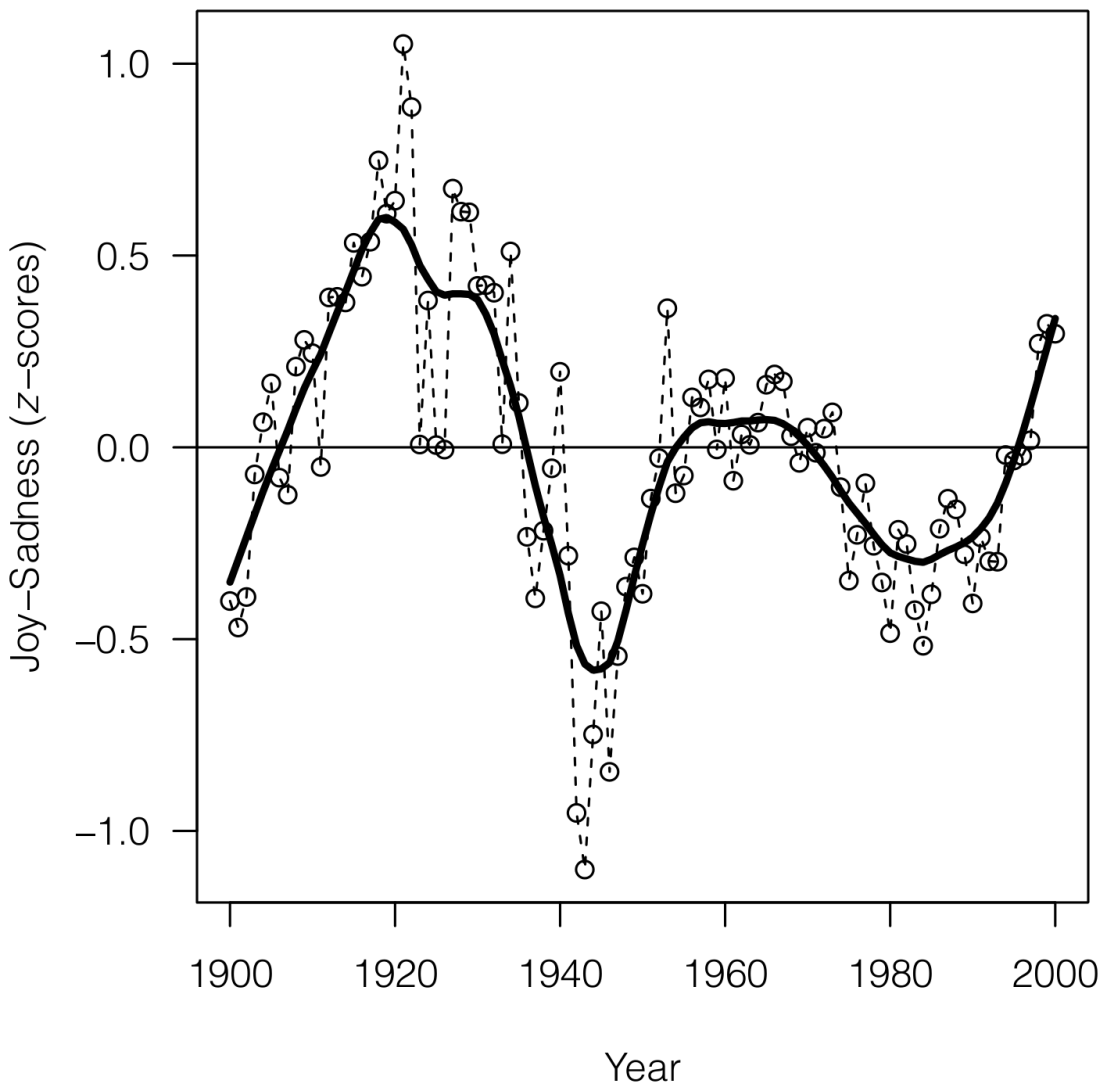
Historical periods of positive and negative moods. Difference between 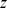-scores of Joy and Sadness for years from 1900 to 2000 (raw data and smoothed trend). Values above zero indicate generally ‘happy’ periods, and values below the zero indicate generally ‘sad’ periods. Values are smoothed using Friedman's ‘super smoother' through R function supsmu() [47].

Our analysis yielded three main results. First, we can distinguish between ‘happy’ and ‘sad’ periods in the data, plotting the differences between 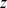-scores (see Methods) for Joy and Sadness in the 1-grams English data set. Figure 1 shows that moods tracked broad historical trends, including a ‘sad’ peak corresponding to Second World War, and two ‘happy’ peaks, one in the 1920's and the other in the 1960's. In more recent years we can see a ‘sad’ period starting from the 1970's, with an increase in ‘happiness’ in the last years of the data set. Interestingly, the First World War does not seem to register a particular change in mood words (Figure 1).

**Figure 2:**
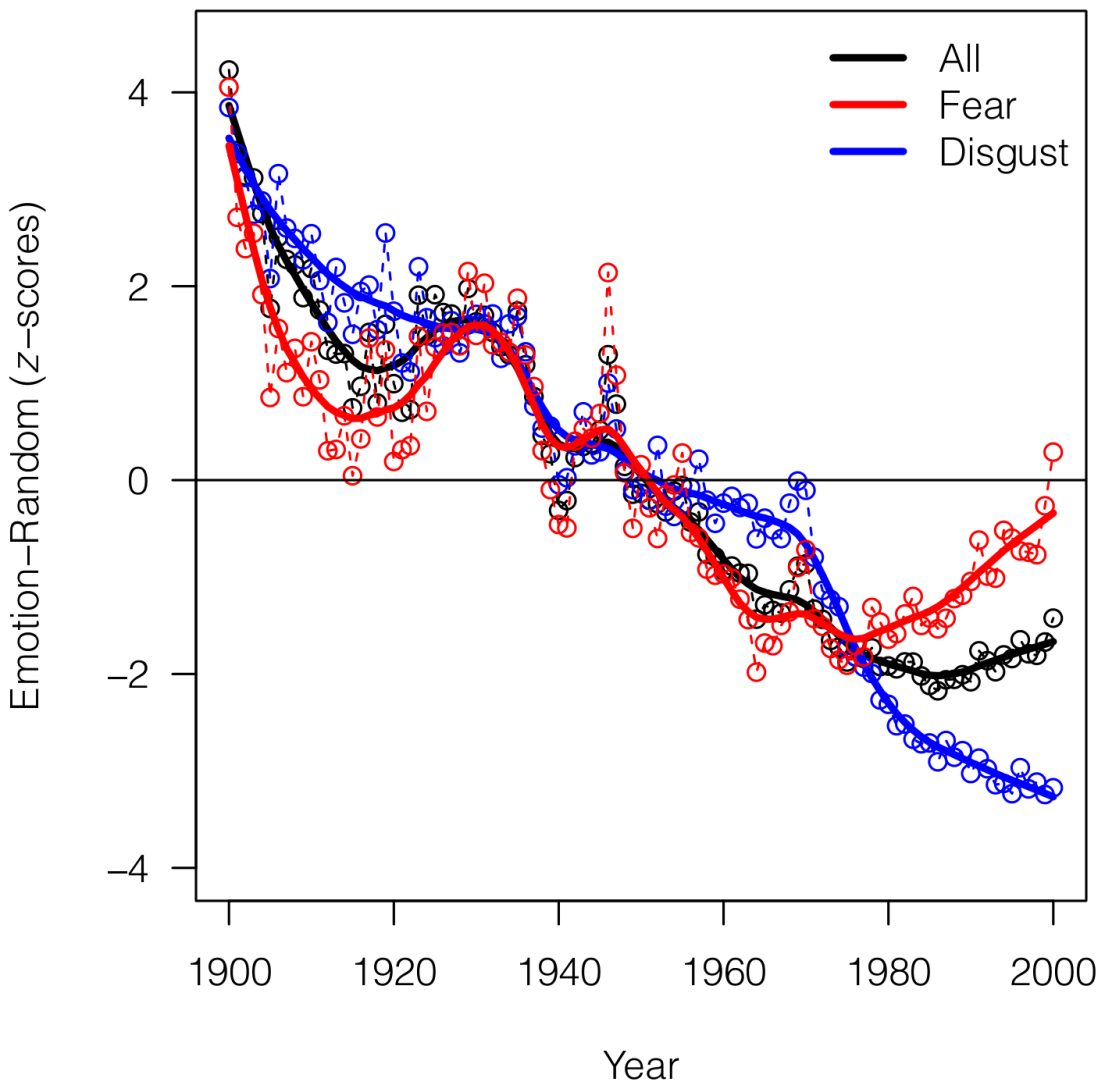
Decrease in the use of emotion-related words through time. Difference between 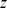-scores of the six emotions and of a random sample of stems (see Methods) for years from 1900 to 2000 (raw data and smoothed trend). Red: the trend for Fear (raw data and smoothed trend), the emotion with the highest final value. Blue: the trend for Disgust (raw data and smoothed trend), the emotion with the lowest final value. Values are smoothed using Friedman's ‘super smoother’ through R function supsmu() [47].

Our second finding is a clear decrease in the overall use of mood words through time (Figure 2). We performed checks to confirm that the overall decrease in mood word frequency in the data is not merely a reflection of, for example, greater numbers of technically-oriented or scientific books through time. Although the Ngram database does not give an explicit breakdown of book subject categories [2], we analyzed the same mood word lists on Google's 1-grams English Fiction data set, which contains only works of fiction and literary criticism. In support of a real decrease in literary emotion, we found a similar decrease in the overall use of mood words (see Figure S1).

Within this general decrease, we identify Disgust as the emotion with the lowest final 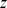-score and Fear as having the highest final 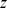-score (Figure 2). Notably, the mood of Fear, which declined throughout most of the early century, has increased markedly since the 1970's, in contrast to the continued decline of other moods (Figure 2).

**Figure 3:**
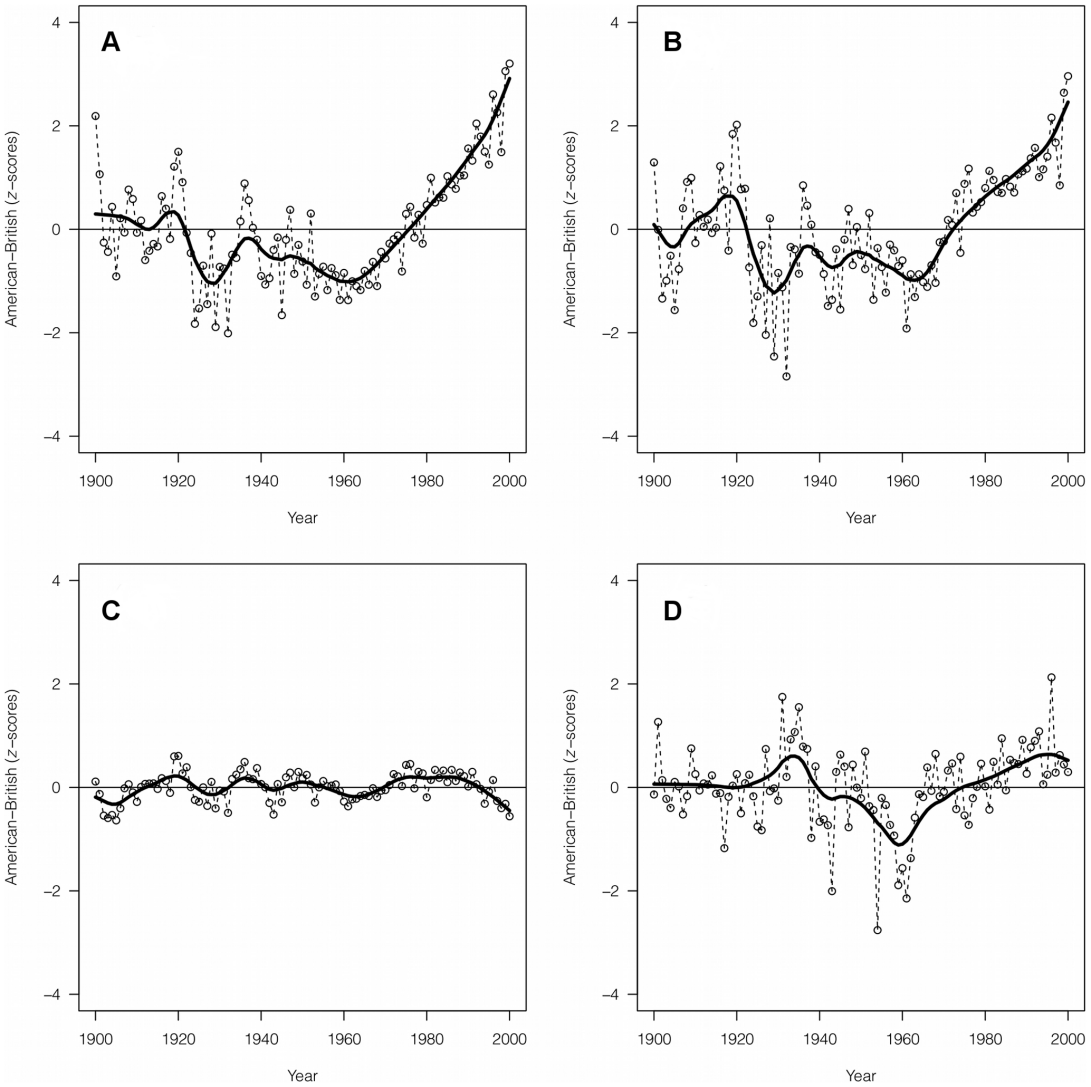
Differences between American English and British English. Difference between 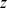-scores in American English and British English for years from 1900 to 2000 (raw data and smoothed trend). A: Emotion terms. B: Content-free words. C: Random sample. D: 100 largest urban agglomerations in the world. Values are smoothed using Friedman's ‘super smoother’ through R function supsmu() [47].

Our third finding is that, since about 1960, American books have increased their mood contents compared to British books. This divergence between American and British English occurs within the context of the overall decline in the use of mood words. If we plot the difference in 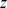-scores between American and British word data (Figure 3a), we see a clear, steady, relative increase in American emotion-related words from 1960 to 2000. Since about 1980, books written in American have been more ‘emotional’ (in all mood figures) than the ones written in British (Figure 3a). This difference in 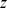-scores – which reflects the respective deviations from each nation's mean value – is duplicated also by the same change in absolute emotion scores (see Methods): American and British English have similar absolute emotion scores in the first half of the 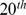 century (or even British slightly more emotional), followed by a relative increase in the emotion scores for just the American English data set (data not shown).

Again to confirm the reality of the pattern, we checked this American–British divergence in emotion words against other indicators. Figure 3b shows the difference between American and British usage of the 307 content-free words compiled by Hughes et al. [7]. The post-1960 divergence in content-free word usage (Figure 3b) is strikingly similar to the divergence in emotional word usage (Figure 3a). As mentioned in the Introduction, changes in the use of content-free words have been associated with broader stylistic differentiations [7], hence our analysis suggests that the divergence between American and British English in respect to the use of mood words is paired by a more general stylistic divergence since the 1960s.

As above, this does not appear to be an artifact of the Ngram data. To test this, we also checked the divergence using a random sample of words (Figure 3c) or in the usage of the names of the 100 most populated cities (Figure 3d) from the same American and British Ngram datasets. Neither showed the same change we see in both emotion words and content-free words. This suggests that emotional content and style were coupled in a distinct way, and that the divergence of these between British and American English stands out from the background of other word types.

## Discussion

Using the extraordinary new data on word frequencies in books [2], we find that significant changes in the usage of more generalized mood terms are also detectable through the years. While studies of online social media have shown how short–term patterns in word usage respond to socio-political events [9], [24]–[26], here we find that the expression of moods in books also reflects much longer–term trends of years or even decades. These changes in literary mood are seemingly driven by major 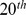 century phenomena such as World War II, The Great Depression, or the Baby Boom.

We also found a general decrease in emotional word usage in the past decades up to the present, which was observed also in fiction writing on its own. We interpret this as a genuine decrease in the literary expression of emotion, but an alternative explanation could be that mood words have changed, rather than decreased in usage, through the 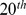 century. This seems unlikely to explain the observed decrease, however, because we used contemporary word lists, analyzed recently in Twitter data to characterize recent events [24], any bias of which should have increased in usage towards the present.

Our results also support the popular notion that American authors express more emotion than the British. Somewhat surprisingly, this difference has apparently developed only since the 1960s, and as part of a more general stylistic differentiation in American versus British English, reflected similarly in content-free word frequencies. This relative increase of American mood word use roughly coincides with the increase of anti–social and narcissistic sentiments in U.S. popular song lyrics from 1980 to 2007 [6], as evidenced by steady increases in angry/antisocial lyrics and in the percentage of first-person singular pronouns (e.g., *I*, *me*, *mine*), with a corresponding decrease in words indicating social interactions (e.g., *mate*, *talk*, *child*) over the same 27-year period [6].

As these findings appear to genuinely reflect changes in published language, a remaining question is whether word usage represents real behavior in a population, or possibly an absence of that behavior which is increasingly played out via literary fiction (or online discourse). It has been suggested, for example, that it was the suppression of desire in ordinary Elizabethan English life that increased demand for writing “obsessed with romance and sex” [27]. So while it is easy to conclude that Americans have themselves become more ‘emotional’ over the past several decades, perhaps songs and books may not reflect the real population any more than catwalk models reflect the average body; the observed changes reflect the book market, rather than a direct change in American culture. We believe the changes do reflect changes in culture, however, because unlike lyrics of the top 10 songs, the book data are independent of book sales [2]. Although authors may not be a perfectly representative subset of the general population, at least the Google dataset is not as overtly commercial as song lyrics or any of the other ubiquitous “most popular” lists of online media. Furthermore, the association of mood changes with major 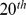 century economic and political events supports the fact that word usage, as retrieved from Google dataset, reveals the long term response to these events in a much broader population of book authors. The dynamics of the feedback between book authors and the wider public can be explored by future studies involving the Ngram dataset.

In any case, changes in culture consist of changes in cultural artifacts, of which words are an informative sample [2], [6]–[8], [28]–[30]. Future studies will surely explore diversity more closely. A population-level mean – including what we have reported here – does not necessarily track a typical behavior, so the meaning of patterns will become refined by addressing changes cross-culturally (e.g. non-English and non-Western languages), and at the smaller community scale [31]. Another promising development is the analysis of more complex sets of cultural traits that might be more diagnostic than mood words or content-free words.

More generally, we hope that we can contribute to the world of Big Data studies by showing that time depth is a crucial dimension. Our results on the long–term, mass scale encourage the more detailed use of word data to characterize the evolution of cultural differences and trends, to detect patterns previously unknown through conventional history [7], [25]. While new theoretical and modelling approaches have rapidly multiplied in the field of cultural evolution (see e.g. [32]–[36]), we believe that the current availability and abundance of quantitative data represents an extraordinary, and much needed, opportunity to provide empirical validation in human cultural dynamics studies.

## Methods

For this study we assessed the emotional valence of the text in books using a text analysis tool, namely WordNet Affect [37]–[39]. WordNet Affect builds on WordNet [40] by labeling synonymous terms which may represent mood states. Six mood categories, each represented by a different number of terms, have been analyzed: Anger (N = 146), Disgust (N = 30), Fear (N = 92), Joy (N = 224), Sadness (N = 115), and Surprise (N = 41). The text analysis was performed on word stems; the latter were formed using Porter's Algorithm [41]. Both WordNet Affect and Porter's Algorithm are considered as standard tools in text mining and have been applied in several relevant tasks [24], [42]–[46]. We obtained the time series of stemmed word frequencies via Google's Ngram tool (http://books.google.com/ngrams/datasets) in four distinct data sets: 1-grams English (combining both British and American English), 1-grams English Fiction (containing only fiction books), 1-grams American English, and 1-grams British English.

For each stemmed word we collected the amount of occurrences (case insensitive) in each year from 1900 to 2000 (both included). We excluded years before 1900 since the number of books before 1900 is considerably lower, and years after 2000 since books published recently are still being included in the data set, and therefore latest records are incomplete and possibly biased. Because the number of books scanned in the data set varies from year to year, to obtain frequencies for performing the analysis we normalized the yearly amount of occurrences using the occurrences, for each year, of the word “the”, which is considered as a reliable indicator of the total number of words in the data set. We preferred to normalize by the word “the”, rather than by the total number of words, to avoid the effect of the influx of data, special characters, etc. that may have come into books recently. The word “the” is about 5–6% of all words, and a good representative of real writing, and real sentences. To test the robustness of the normalization, we also performed the same analysis reported in Figure 1 (differences between 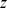-scores (see below) for Joy and Sadness in the 1-grams English data set) using two alternative normalizations, namely the cumulative count of the top 10 most frequent words each year (Figure S2a), and the total counts of 1-grams as in [2] (Figure S2b). The resulting time series are higly correlated (see the legend of Figure S2), confirming the robustness of the normalization.

For a year 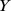, given the count 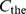 of the word “the” in the corpus as well as the counts 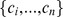 of the 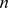 WordNet terms representing one of the six considered mood types, we computed a mood score (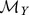) as follows:
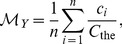
(1)i.e. a mood score is essentially the average normalized frequency across the considered mood terms. In order to compare different types of moods effectively, after computing the mood scores for the entire set of years (1900 to 2000), we converted them to their 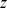-score equivalent (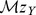), using:
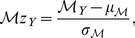
(2)where 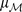 and 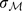 denote the mean and standard deviation of the mood scores across the considered set of years.

When determining the ‘absolute’ trends of moods (results in Figure 2), to avoid any possible bias related to normalization, we compared the 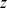-scores of moods time series with a 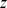-score derived by a random sample of stems. This random sample was obtained by extracting 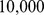 random terms from the Part of Speech database, which is a combination of Moby Part-of-Speech II and WordNet database, and contains 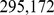 terms (http://wordlist.sourceforge.net/pos-readme). The terms were stemmed using Porter's Algorithm [41]. Duplicated stems and stems shorter then two letters were finally eliminated obtaining a sample of 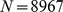 stems that we used for the analysis.

Finally (results in Figure 3), we compared the difference between American English and British English books in three other data sets (in addition to mood terms): content-free words, using the list of the 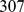 content-free words provided by Huges et al. [7], the same random stems used for Figure 2, and the list of the 100 largest urban agglomerations in the world (from http://en.wikipedia.org/wiki/List_of_urban_agglomerations_by_population_(United_Nations), containing terms like *Tokyo*, *Karachi*, and *Berlin*. Agglomerations with composite names, e.g. *New York*–*Newark*, *Rio de Janeiro* were excluded).

## Supporting Information

Figure S1
**Decrease in the use of emotion-related words through time in fiction books.** Difference between 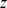-scores of the six emotions and of a random sample of stems (see Methods) for years from 1900 to 2000 (raw data and smoothed trend) in the 1-grams English Fiction data set. Red: the trend for Fear (raw data and smoothed trend), the emotion with the highest final value. Blue: the trend for Disgust (raw data and smoothed trend), the emotion with the lowest final value. Values are smoothed using Friedman's ‘super smoother’ through R function supsmu() [47].(TIFF)

Figure S2
**Historical periods of positive and negative moods with alternative normalizations.** Difference between 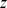-scores of Joy and Sadness for years from 1900 to 2000 (raw data and smoothed trend). Values above zero indicate generally ‘happy’ periods, and values below the zero indicate generally ‘sad’ periods. Values are smoothed using Friedman's ‘super smoother’ through R function supsmu() [47]. A: Frequencies are normalized using the cumulative count of the top 10 most frequent words for each year. Correlations with the time series used in the analysis (normalized with the yearly count of “the”) are statistically significant (Pearson's 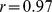 for row data, and 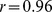 for smoothed data. In both cases 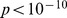 and 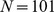). B: Frequencies are normalized using the total counts of 1-grams for each year. Also in this case correlations with the time series used in the analysis are statistically significant (Pearson's 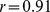 for row data, and 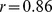 for smoothed data. In both cases 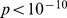 and 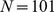).(TIFF)
