# Supplementary figures and images for: The Expression of Emotions in 20th Century Books
Source: PLoS One. 2013 Mar 20;8(3):e59030. doi: 10.1371/journal.pone.0059030 (PMC3604170; doi:10.1371/journal.pone.0059030)

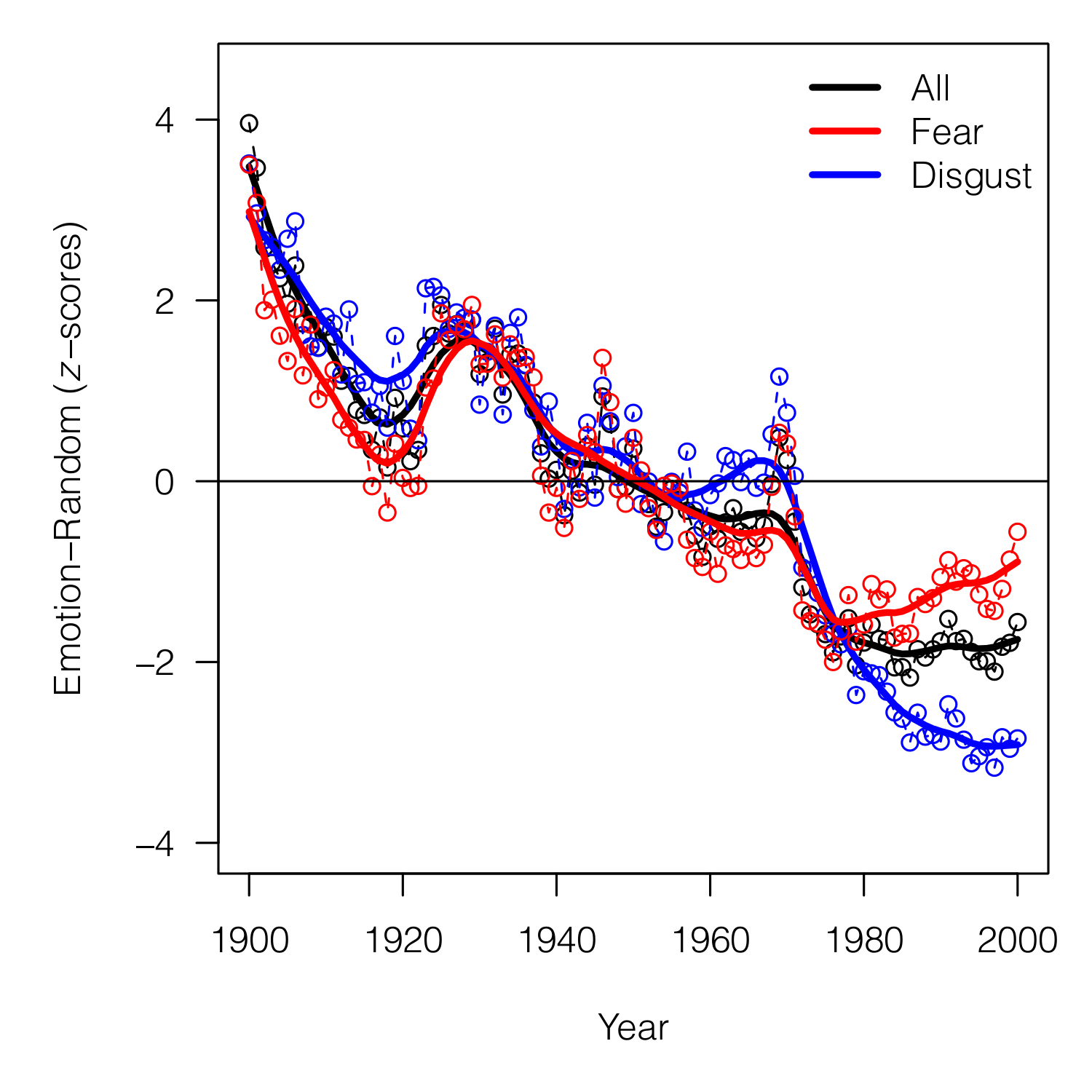

Supplement: Figure S1 — Decrease in the use of emotion-related words through time in fiction books. Difference between -scores of the six emotions and of a random sample of stems (see Methods) for years from 1900 to 2000 (raw data and smoothed trend) in the 1-grams English Fiction data set. Red: the trend for Fear (raw data and smoothed trend), the emotion with the highest final value. Blue: the trend for Disgust (raw data and smoothed trend), the emotion with the lowest final value. Values are smoothed using Friedman's ‘super smoother’ through R function supsmu() [47]. (TIFF) [file pone.0059030.s001.tiff]

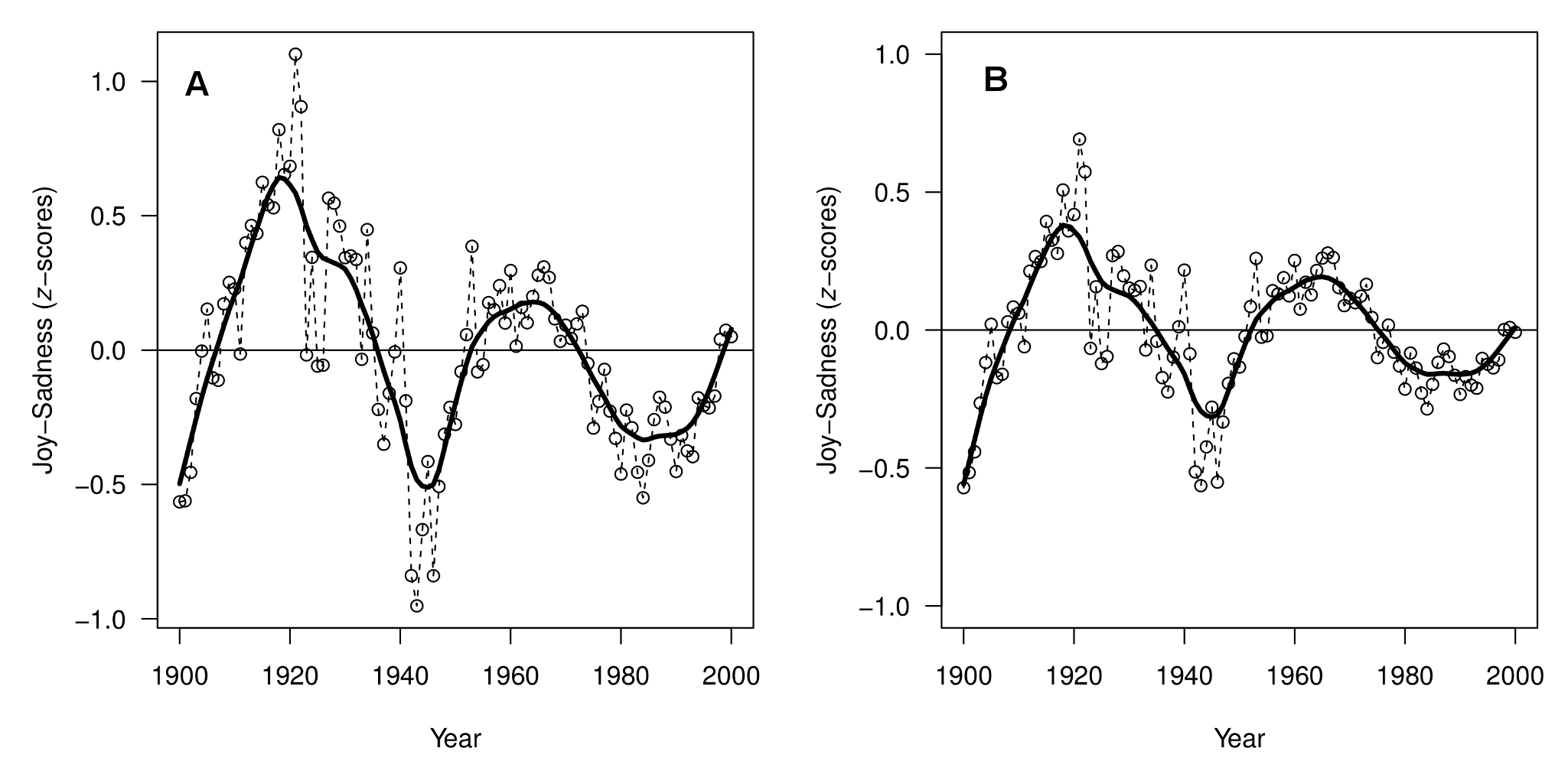

Supplement: Figure S2 — Historical periods of positive and negative moods with alternative normalizations. Difference between -scores of Joy and Sadness for years from 1900 to 2000 (raw data and smoothed trend). Values above zero indicate generally ‘happy’ periods, and values below the zero indicate generally ‘sad’ periods. Values are smoothed using Friedman's ‘super smoother’ through R function supsmu() [47]. A: Frequencies are normalized using the cumulative count of the top 10 most frequent words for each year. Correlations with the time series used in the analysis (normalized with the yearly count of “the”) are statistically significant (Pearson's for row data, and for smoothed data. In both cases and ). B: Frequencies are normalized using the total counts of 1-grams for each year. Also in this case correlations with the time series used in the analysis are statistically significant (Pearson's for row data, and for smoothed data. In both cases and ). (TIFF) [file pone.0059030.s002.tiff]
